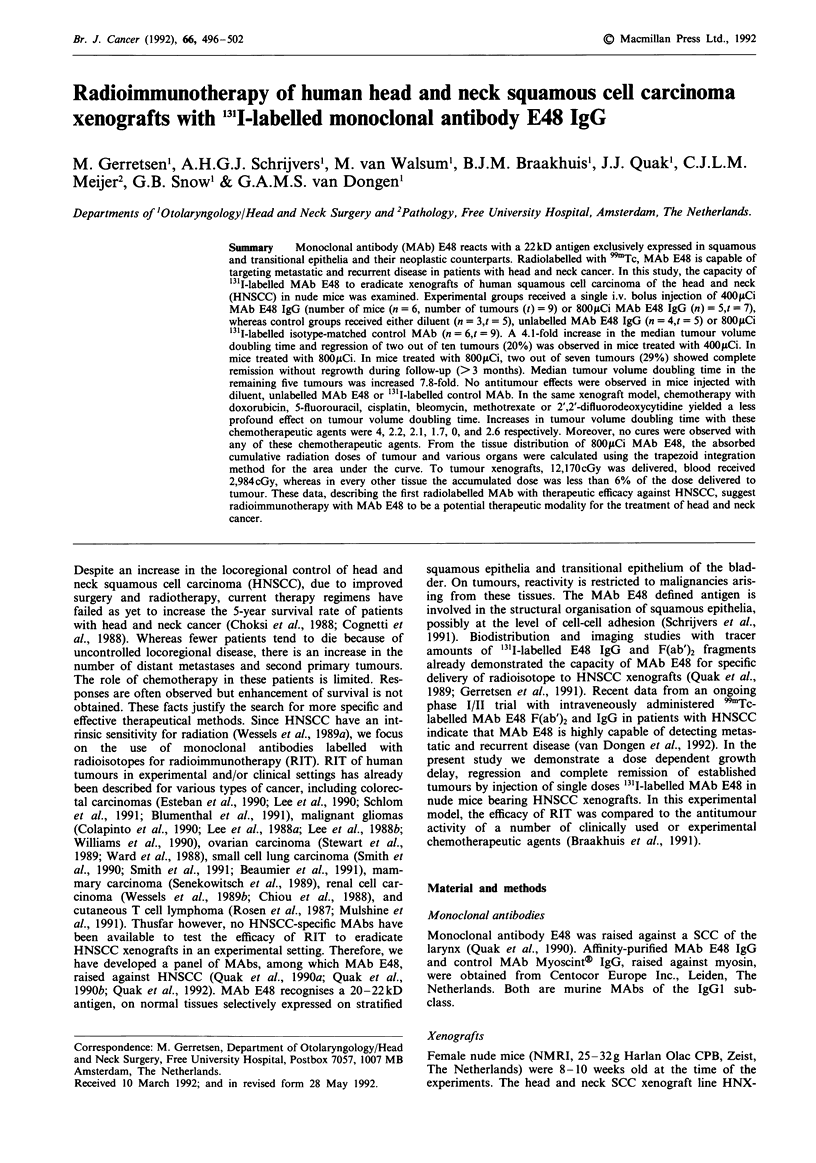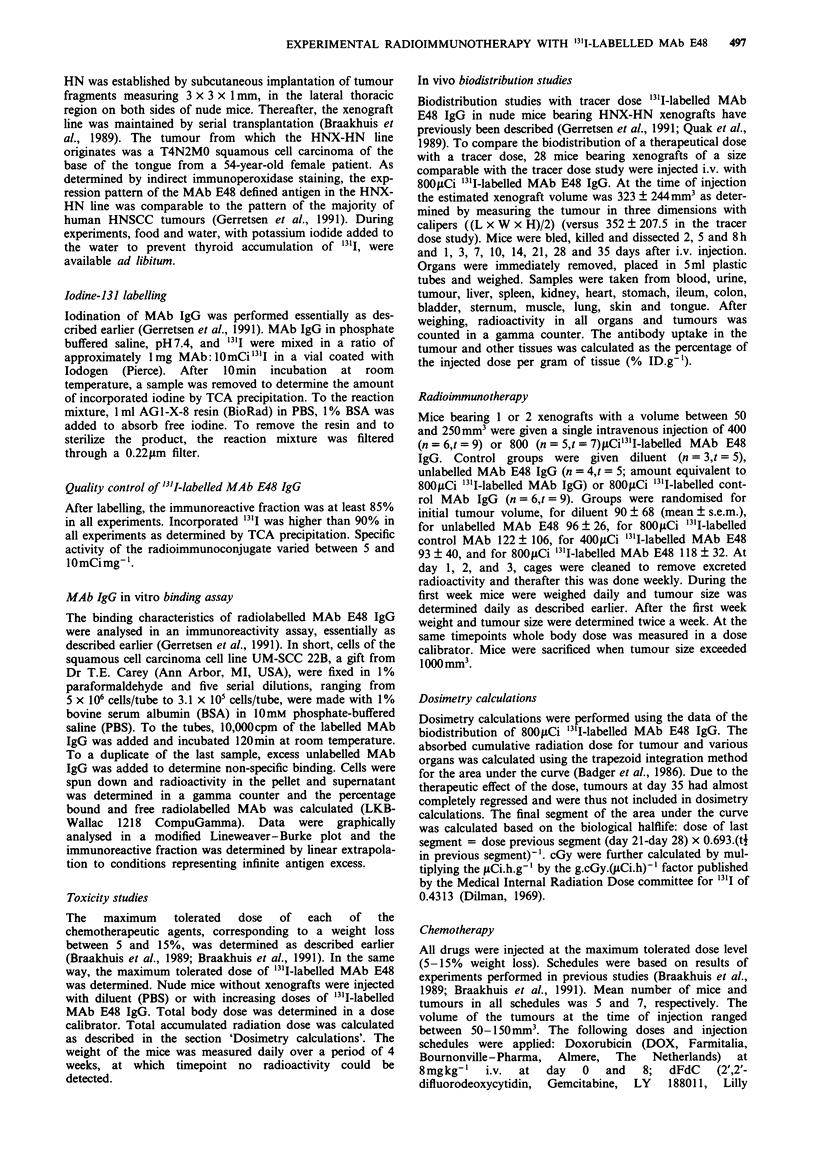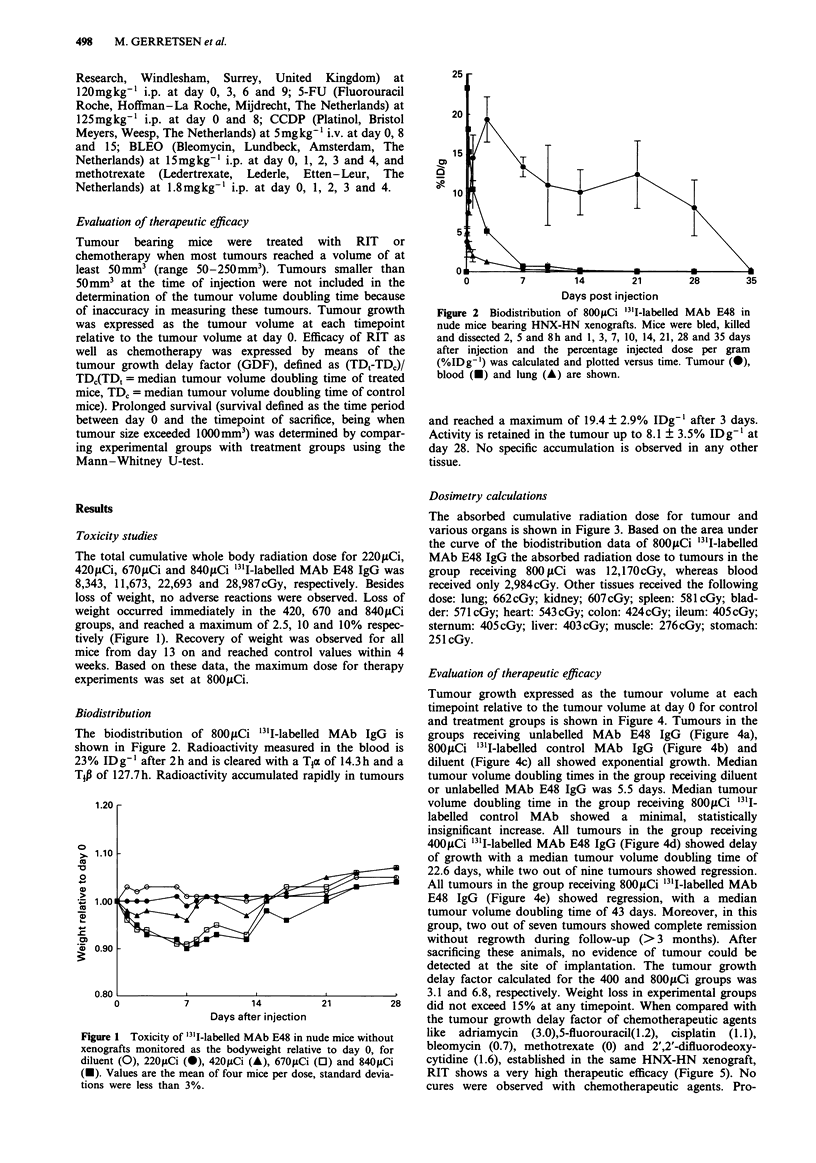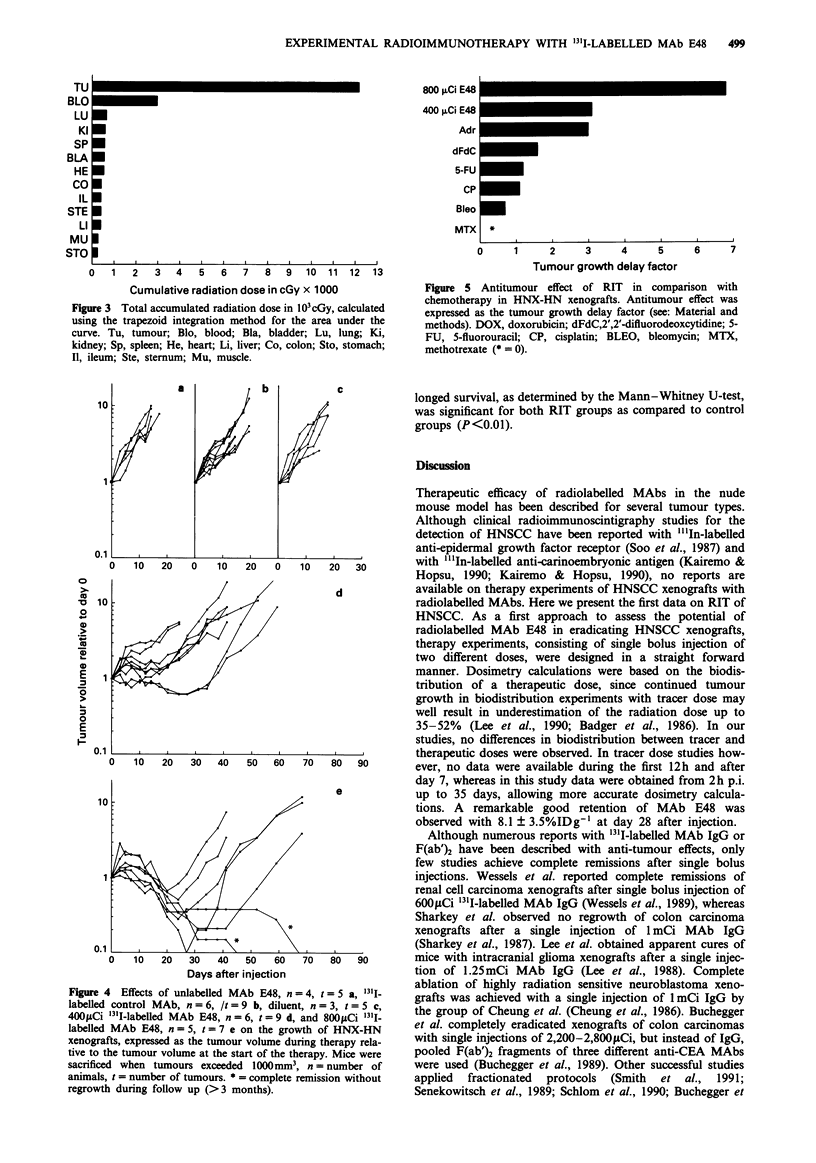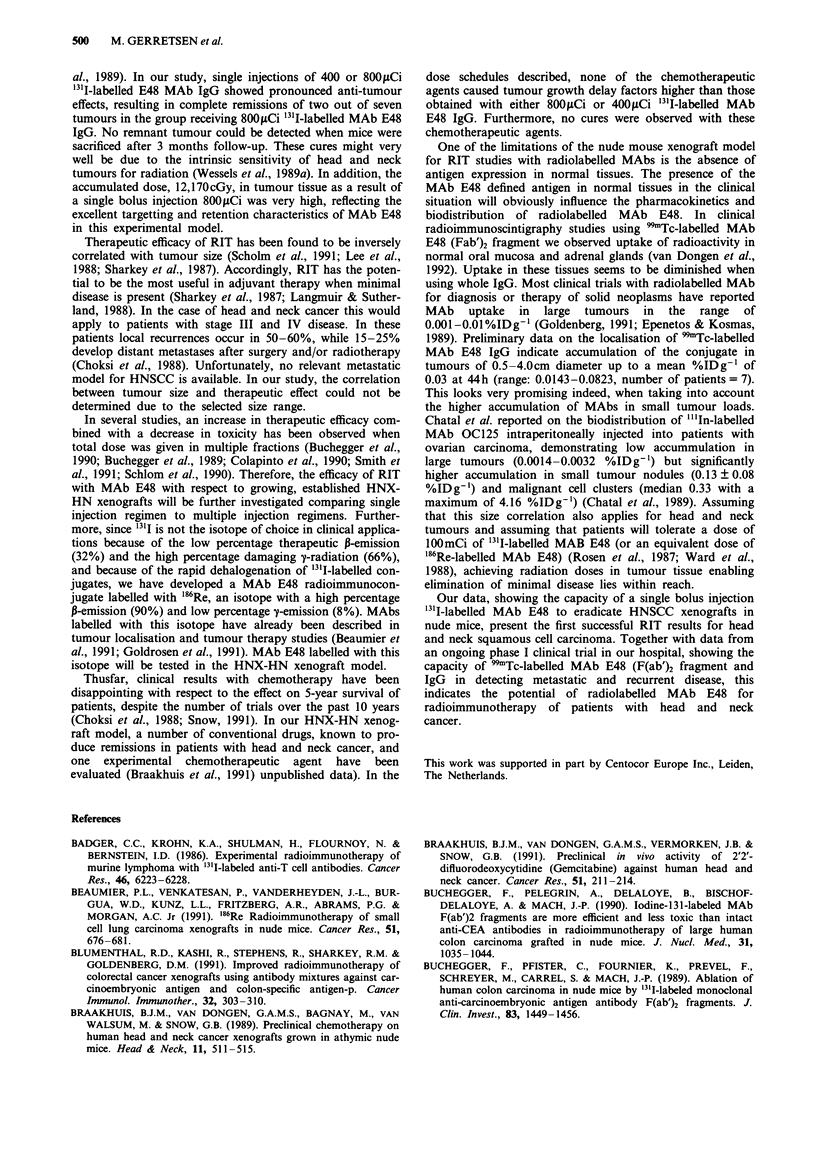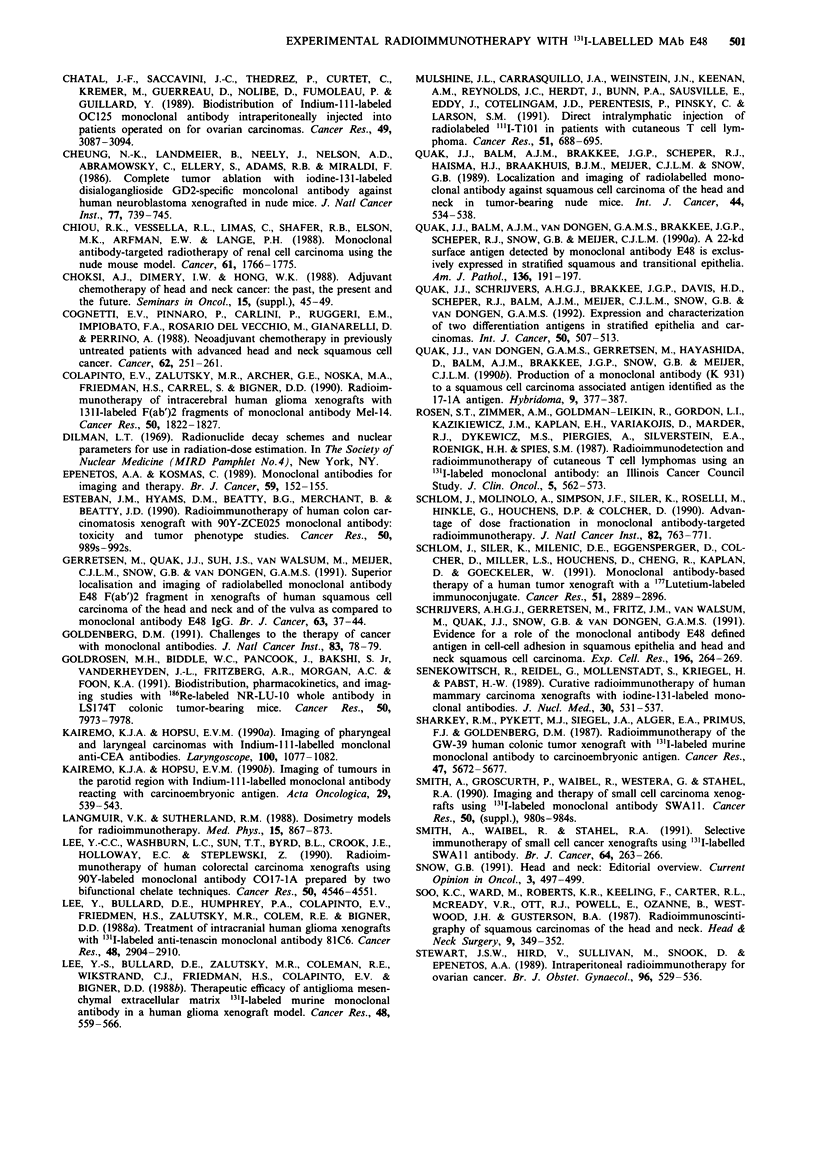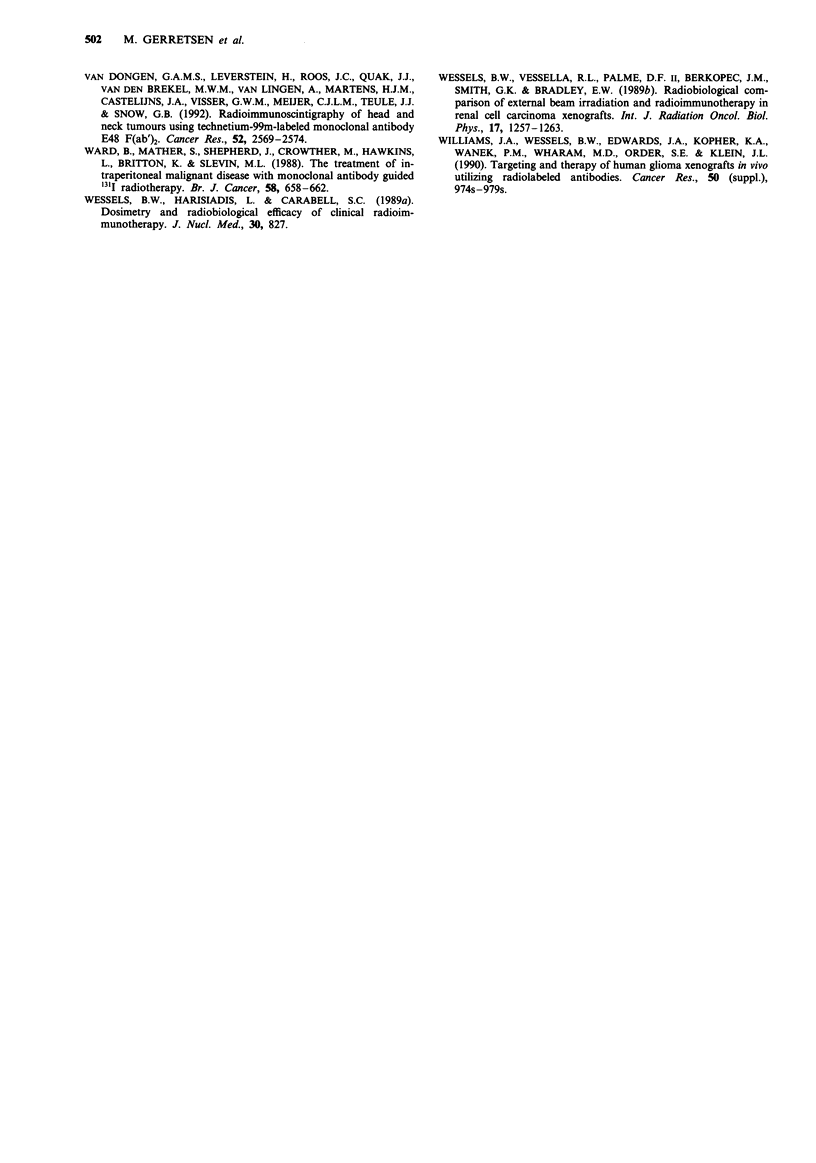# Radioimmunotherapy of human head and neck squamous cell carcinoma xenografts with 131I-labelled monoclonal antibody E48 IgG.

**DOI:** 10.1038/bjc.1992.302

**Published:** 1992-09

**Authors:** M. Gerretsen, A. H. Schrijvers, M. van Walsum, B. J. Braakhuis, J. J. Quak, C. J. Meijer, G. B. Snow, G. A. van Dongen

**Affiliations:** Department of Otolaryngology/Head and Neck Surgery, Free University Hospital, Amsterdam, The Netherlands.

## Abstract

Monoclonal antibody (MAb) E48 reacts with a 22 kD antigen exclusively expressed in squamous and transitional epithelia and their neoplastic counterparts. Radiolabelled with 99mTc, MAb E48 is capable of targeting metastatic and recurrent disease in patients with head and neck cancer. In this study, the capacity of 131I-labelled MAb E48 to eradicate xenografts of human squamous cell carcinoma of the head and neck (HNSCC) in nude mice was examined. Experimental groups received a single i.v. bolus injection of 400 microCi MAb E48 IgG (number of mice (n = 6, number of tumours (t) = 9) or 800 microCi MAb E48 IgG (n) = 5,t = 7), whereas control groups received either diluent (n = 3,t = 5), unlabelled MAb E48 IgG (n = 4,t = 5) or 800 microCi 131I-labelled isotype-matched control MAb (n = 6,t = 9). A 4.1-fold increase in the median tumour volume doubling time and regression of two out of ten tumours (20%) was observed in mice treated with 400 microCi. In mice treated with 800 microCi. In mice treated with 800 microCi, two out of seven tumours (29%) showed complete remission without regrowth during follow-up (greater than 3 months). Median tumour volume doubling time in the remaining five tumours was increased 7.8-fold. No antitumour effects were observed in mice injected with diluent, unlabelled MAb E48 or 131I-labelled control MAb. In the same xenograft model, chemotherapy with doxorubicin, 5-fluorouracil, cisplatin, bleomycin, methotrexate or 2',2'-difluorodeoxycytidine yielded a less profound effect on tumour volume doubling time. Increases in tumour volume doubling time with these chemotherapeutic agents were 4, 2.2, 2.1, 1.7, 0, and 2.6 respectively. Moreover, no cures were observed with any of these chemotherapeutic agents. From the tissue distribution of 800 microCi MAb E48, the absorbed cumulative radiation doses of tumour and various organs were calculated using the trapezoid integration method for the area under the curve. To tumour xenografts, 12,170 cGy was delivered, blood received 2,984 cGy, whereas in every other tissue the accumulated dose was less than 6% of the dose delivered to tumour. These data, describing the first radiolabelled MAb with therapeutic efficacy against HNSCC, suggest radioimmunotherapy with MAb E48 to be a potential therapeutic modality for the treatment of head and neck cancer.


*Br. J. Cancer* (1992), **66**, 496–502 © Macmillan Press Ltd., 1992

### Radioimmunotherapy of human head and neck squamous cell carcinoma xenografts with $^{131}$I-labelled monoclonal antibody E48 IgG

M. Gerretsen[1], A.H.G.J. Schrijvers[1], M. van Walsum[1], B.J.M. Braakhuis[1], J.J. Quak[1], C.J.L.M. Meijer[2], G.B. Snow[1] & G.A.M.S. van Dongen[1]

*Departments of [1]Otolaryngology/Head and Neck Surgery and [2]Pathology, Free University Hospital, Amsterdam, The Netherlands.*

**Summary** Monoclonal antibody (MAb) E48 reacts with a 22 kD antigen exclusively expressed in squamous and transitional epithelia and their neoplastic counterparts. Radiolabelled with $^{99m}$Tc, MAb E48 is capable of targeting metastatic and recurrent disease in patients with head and neck cancer. In this study, the capacity of $^{131}$I-labelled MAb E48 to eradicate xenografts of human squamous cell carcinoma of the head and neck (HNSCC) in nude mice was examined. Experimental groups received a single i.v. bolus injection of 400 µCi MAb E48 IgG (number of mice ($n = 6$, number of tumours ($t$) = 9) or 800 µCi MAb E48 IgG ($n$) = 5,$t$ = 7), whereas control groups received either diluent ($n = 3, t = 5$), unlabelled MAb E48 IgG ($n = 4, t = 5$) or 800 µCi $^{131}$I-labelled isotype-matched control MAb ($n = 6, t = 9$). A 4.1-fold increase in the median tumour volume doubling time and regression of two out of ten tumours (20%) was observed in mice treated with 400 µCi. In mice treated with 800 µCi. In mice treated with 800 µCi, two out of seven tumours (29%) showed complete remission without regrowth during follow-up (>3 months). Median tumour volume doubling time in the remaining five tumours was increased 7.8-fold. No antitumour effects were observed in mice injected with diluent, unlabelled MAb E48 or $^{131}$I-labelled control MAb. In the same xenograft model, chemotherapy with doxorubicin, 5-fluorouracil, cisplatin, bleomycin, methotrexate or 2',2'-difluorodeoxycytidine yielded a less profound effect on tumour volume doubling time. Increases in tumour volume doubling time with these chemotherapeutic agents were 4, 2.2, 2.1, 1.7, 0, and 2.6 respectively. Moreover, no cures were observed with any of these chemotherapeutic agents. From the tissue distribution of 800 µCi MAb E48, the absorbed cumulative radiation doses of tumour and various organs were calculated using the trapezoid integration method for the area under the curve. To tumour xenografts, 12,170 cGy was delivered, blood received 2,984 cGy, whereas in every other tissue the accumulated dose was less than 6% of the dose delivered to tumour. These data, describing the first radiolabelled MAb with therapeutic efficacy against HNSCC, suggest radioimmunotherapy with MAb E48 to be a potential therapeutic modality for the treatment of head and neck cancer.

Despite an increase in the locoregional control of head and neck squamous cell carcinoma (HNSCC), due to improved surgery and radiotherapy, current therapy regimens have failed as yet to increase the 5-year survival rate of patients with head and neck cancer (Choksi *et al.*, 1988; Cognetti *et al.*, 1988). Whereas fewer patients tend to die because of uncontrolled locoregional disease, there is an increase in the number of distant metastases and second primary tumours. The role of chemotherapy in these patients is limited. Responses are often observed but enhancement of survival is not obtained. These facts justify the search for more specific and effective therapeutical methods. Since HNSCC have an intrinsic sensitivity for radiation (Wessels *et al.*, 1989*a*), we focus on the use of monoclonal antibodies labelled with radioisotopes for radioimmunotherapy (RIT). RIT of human tumours in experimental and/or clinical settings has already been described for various types of cancer, including colorectal carcinomas (Esteban *et al.*, 1990; Lee *et al.*, 1990; Schlom *et al.*, 1991; Blumenthal *et al.*, 1991), malignant gliomas (Colapinto *et al.*, 1990; Lee *et al.*, 1988*a*; Lee *et al.*, 1988*b*; Williams *et al.*, 1990), ovarian carcinoma (Stewart *et al.*, 1989; Ward *et al.*, 1988), small cell lung carcinoma (Smith *et al.*, 1990; Smith *et al.*, 1991; Beaumier *et al.*, 1991), mammary carcinoma (Senekowitsch *et al.*, 1989), renal cell carcinoma (Wessels *et al.*, 1989*b*; Chiou *et al.*, 1988), and cutaneous T cell lymphoma (Rosen *et al.*, 1987; Mulshine *et al.*, 1991). Thusfar however, no HNSCC-specific MAbs have been available to test the efficacy of RIT to eradicate HNSCC xenografts in an experimental setting. Therefore, we have developed a panel of MAbs, among which MAb E48, raised against HNSCC (Quak *et al.*, 1990*a*; Quak *et al.*, 1990*b*; Quak *et al.*, 1992). MAb E48 recognises a 20–22 kD antigen, on normal tissues selectively expressed on stratified squamous epithelia and transitional epithelium of the bladder. On tumours, reactivity is restricted to malignancies arising from these tissues. The MAb E48 defined antigen is involved in the structural organisation of squamous epithelia, possibly at the level of cell-cell adhesion (Schrijvers *et al.*, 1991). Biodistribution and imaging studies with tracer amounts of $^{131}$I-labelled E48 IgG and F(ab')$_2$ fragments already demonstrated the capacity of MAb E48 for specific delivery of radioisotope to HNSCC xenografts (Quak *et al.*, 1989; Gerretsen *et al.*, 1991). Recent data from an ongoing phase I/II trial with intraveneously administered $^{99m}$Tc-labelled MAb E48 F(ab')$_2$ and IgG in patients with HNSCC indicate that MAb E48 is highly capable of detecting metastatic and recurrent disease (van Dongen *et al.*, 1992). In the present study we demonstrate a dose dependent growth delay, regression and complete remission of established tumours by injection of single doses $^{131}$I-labelled MAb E48 in nude mice bearing HNSCC xenografts. In this experimental model, the efficacy of RIT was compared to the antitumour activity of a number of clinically used or experimental chemotherapeutic agents (Braakhuis *et al.*, 1991).

### Material and methods

### *Monoclonal antibodies*

Monoclonal antibody E48 was raised against a SCC of the larynx (Quak *et al.*, 1990). Affinity-purified MAb E48 IgG and control MAb Myoscint® IgG, raised against myosin, were obtained from Centocor Europe Inc., Leiden, The Netherlands. Both are murine MAbs of the IgG1 subclass.

### *Xenografts*

Female nude mice (NMRI, 25–32 g Harlan Olac CPB, Zeist, The Netherlands) were 8–10 weeks old at the time of the experiments. The head and neck SCC xenograft line HNX-

Correspondence: M. Gerretsen, Department of Otolaryngology/Head and Neck Surgery, Free University Hospital, Postbox 7057, 1007 MB Amsterdam, The Netherlands.
Received 10 March 1992; and in revised form 28 May 1992.

HN was established by subcutaneous implantation of tumour fragments measuring 3 × 3 × 1 mm, in the lateral thoracic region on both sides of nude mice. Thereafter, the xenograft line was maintained by serial transplantation (Braakhuis *et al.*, 1989). The tumour from which the HNX-HN line originates was a T4N2M0 squamous cell carcinoma of the base of the tongue from a 54-year-old female patient. As determined by indirect immunoperoxidase staining, the expression pattern of the MAb E48 defined antigen in the HNX-HN line was comparable to the pattern of the majority of human HNSCC tumours (Gerretsen *et al.*, 1991). During experiments, food and water, with potassium iodide added to the water to prevent thyroid accumulation of $^{131}$I, were available *ad libitum*.

*Iodine-131 labelling*

Iodination of MAb IgG was performed essentially as described earlier (Gerretsen *et al.*, 1991). MAb IgG in phosphate buffered saline, pH 7.4, and $^{131}$I were mixed in a ratio of approximately 1 mg MAb: 10 mCi $^{131}$I in a vial coated with Iodogen (Pierce). After 10 min incubation at room temperature, a sample was removed to determine the amount of incorporated iodine by TCA precipitation. To the reaction mixture, 1 ml AG1-X-8 resin (BioRad) in PBS, 1% BSA was added to absorb free iodine. To remove the resin and to sterilize the product, the reaction mixture was filtered through a 0.22 μm filter.

*Quality control of $^{131}$I-labelled MAb E48 IgG*

After labelling, the immunoreactive fraction was at least 85% in all experiments. Incorporated $^{131}$I was higher than 90% in all experiments as determined by TCA precipitation. Specific activity of the radioimmunoconjugate varied between 5 and 10 mCi mg$^{-1}$.

*MAb IgG* in vitro *binding assay*

The binding characteristics of radiolabelled MAb E48 IgG were analysed in an immunoreactivity assay, essentially as described earlier (Gerretsen *et al.*, 1991). In short, cells of the squamous cell carcinoma cell line UM-SCC 22B, a gift from Dr T.E. Carey (Ann Arbor, MI, USA), were fixed in 1% paraformaldehyde and five serial dilutions, ranging from $5 \times 10^6$ cells/tube to $3.1 \times 10^5$ cells/tube, were made with 1% bovine serum albumin (BSA) in 10 mM phosphate-buffered saline (PBS). To the tubes, 10,000 cpm of the labelled MAb IgG was added and incubated 120 min at room temperature. To a duplicate of the last sample, excess unlabelled MAb IgG was added to determine non-specific binding. Cells were spun down and radioactivity in the pellet and supernatant was determined in a gamma counter and the percentage bound and free radiolabelled MAb was calculated (LKB-Wallac 1218 CompuGamma). Data were graphically analysed in a modified Lineweaver–Burke plot and the immunoreactive fraction was determined by linear extrapolation to conditions representing infinite antigen excess.

*Toxicity studies*

The maximum tolerated dose of each of the chemotherapeutic agents, corresponding to a weight loss between 5 and 15%, was determined as described earlier (Braakhuis *et al.*, 1989; Braakhuis *et al.*, 1991). In the same way, the maximum tolerated dose of $^{131}$I-labelled MAb E48 was determined. Nude mice without xenografts were injected with diluent (PBS) or with increasing doses of $^{131}$I-labelled MAb E48 IgG. Total body dose was determined in a dose calibrator. Total accumulated radiation dose was calculated as described in the section 'Dosimetry calculations'. The weight of the mice was measured daily over a period of 4 weeks, at which timepoint no radioactivity could be detected.

In vivo *biodistribution studies*

Biodistribution studies with tracer dose $^{131}$I-labelled MAb E48 IgG in nude mice bearing HNX-HN xenografts have previously been described (Gerretsen *et al.*, 1991; Quak *et al.*, 1989). To compare the biodistribution of a therapeutical dose with a tracer dose, 28 mice bearing xenografts of a size comparable with the tracer dose study were injected i.v. with 800 μCi $^{131}$I-labelled MAb E48 IgG. At the time of injection the estimated xenograft volume was 323 ± 244 mm$^3$ as determined by measuring the tumour in three dimensions with calipers ((L × W × H)/2) (versus 352 ± 207.5 in the tracer dose study). Mice were bled, killed and dissected 2, 5 and 8 h and 1, 3, 7, 10, 14, 21, 28 and 35 days after i.v. injection. Organs were immediately removed, placed in 5 ml plastic tubes and weighed. Samples were taken from blood, urine, tumour, liver, spleen, kidney, heart, stomach, ileum, colon, bladder, sternum, muscle, lung, skin and tongue. After weighing, radioactivity in all organs and tumours was counted in a gamma counter. The antibody uptake in the tumour and other tissues was calculated as the percentage of the injected dose per gram of tissue (% ID.g$^{-1}$).

*Radioimmunotherapy*

Mice bearing 1 or 2 xenografts with a volume between 50 and 250 mm$^3$ were given a single intravenous injection of 400 ($n = 6, t = 9$) or 800 ($n = 5, t = 7$) μCi $^{131}$I-labelled MAb E48 IgG. Control groups were given diluent ($n = 3, t = 5$), unlabelled MAb E48 IgG ($n = 4, t = 5$; amount equivalent to 800 μCi $^{131}$I-labelled MAb IgG) or 800 μCi $^{131}$I-labelled control MAb IgG ($n = 6, t = 9$). Groups were randomised for initial tumour volume, for diluent 90 ± 68 (mean ± s.e.m.), for unlabelled MAb E48 96 ± 26, for 800 μCi $^{131}$I-labelled control MAb 122 ± 106, for 400 μCi $^{131}$I-labelled MAb E48 93 ± 40, and for 800 μCi $^{131}$I-labelled MAb E48 118 ± 32. At day 1, 2, and 3, cages were cleaned to remove excreted radioactivity and therafter this was done weekly. During the first week mice were weighed daily and tumour size was determined daily as described earlier. After the first week weight and tumour size were determined twice a week. At the same timepoints whole body dose was measured in a dose calibrator. Mice were sacrificed when tumour size exceeded 1000 mm$^3$.

*Dosimetry calculations*

Dosimetry calculations were performed using the data of the biodistribution of 800 μCi $^{131}$I-labelled MAb E48 IgG. The absorbed cumulative radiation dose for tumour and various organs was calculated using the trapezoid integration method for the area under the curve (Badger *et al.*, 1986). Due to the therapeutic effect of the dose, tumours at day 35 had almost completely regressed and were thus not included in dosimetry calculations. The final segment of the area under the curve was calculated based on the biological halflife: dose of last segment = dose previous segment (day 21-day 28) × 0.693.($t_{\frac{1}{2}}$ in previous segment)$^{-1}$. cGy were further calculated by multiplying the μCi.h.g$^{-1}$ by the g.cGy.(μCi.h)$^{-1}$ factor published by the Medical Internal Radiation Dose committee for $^{131}$I of 0.4313 (Dilman, 1969).

*Chemotherapy*

All drugs were injected at the maximum tolerated dose level (5–15% weight loss). Schedules were based on results of experiments performed in previous studies (Braakhuis *et al.*, 1989; Braakhuis *et al.*, 1991). Mean number of mice and tumours in all schedules was 5 and 7, respectively. The volume of the tumours at the time of injection ranged between 50–150 mm$^3$. The following doses and injection schedules were applied: Doxorubicin (DOX, Farmitalia, Bournonville-Pharma, Almere, The Netherlands) at 8 mg kg$^{-1}$ i.v. at day 0 and 8; dFdC (2′,2′-difluorodeoxycytidin, Gemcitabine, LY 188011, Lilly

Research, Windlesham, Surrey, United Kingdom) at $120 mg kg^{-1}$ i.p. at day 0, 3, 6 and 9; 5-FU (Fluorouracil Roche, Hoffman-La Roche, Mijdrecht, The Netherlands) at $125 mg kg^{-1}$ i.p. at day 0 and 8; CCDP (Platinol, Bristol Meyers, Weesp, The Netherlands) at $5 mg kg^{-1}$ i.v. at day 0, 8 and 15; BLEO (Bleomycin, Lundbeck, Amsterdam, The Netherlands) at $15 mg kg^{-1}$ i.p. at day 0, 1, 2, 3 and 4, and methotrexate (Ledertrexate, Lederle, Etten-Leur, The Netherlands) at $1.8 mg kg^{-1}$ i.p. at day 0, 1, 2, 3 and 4.

*Evaluation of therapeutic efficacy*

Tumour bearing mice were treated with RIT or chemotherapy when most tumours reached a volume of at least $50 mm^3$ (range $50-250 mm^3$). Tumours smaller than $50 mm^3$ at the time of injection were not included in the determination of the tumour volume doubling time because of inaccuracy in measuring these tumours. Tumour growth was expressed as the tumour volume at each timepoint relative to the tumour volume at day 0. Efficacy of RIT as well as chemotherapy was expressed by means of the tumour growth delay factor (GDF), defined as $(TD_t-TD_c)/TD_c$ ($TD_t$ = median tumour volume doubling time of treated mice, $TD_c$ = median tumour volume doubling time of control mice). Prolonged survival (survival defined as the time period between day 0 and the timepoint of sacrifice, being when tumour size exceeded $1000 mm^3$) was determined by comparing experimental groups with treatment groups using the Mann-Whitney U-test.

### Results

*Toxicity studies*

The total cumulative whole body radiation dose for 220μCi, 420μCi, 670μCi and 840μCi $^{131}$I-labelled MAb E48 IgG was 8,343, 11,673, 22,693 and 28,987cGy, respectively. Besides loss of weight, no adverse reactions were observed. Loss of weight occurred immediately in the 420, 670 and 840μCi groups, and reached a maximum of 2.5, 10 and 10% respectively (Figure 1). Recovery of weight was observed for all mice from day 13 on and reached control values within 4 weeks. Based on these data, the maximum dose for therapy experiments was set at 800μCi.

*Biodistribution*

The biodistribution of 800μCi $^{131}$I-labelled MAb IgG is shown in Figure 2. Radioactivity measured in the blood is 23% $IDg^{-1}$ after 2h and is cleared with a $T_{\frac{1}{2}}\alpha$ of 14.3h and a $T_{\frac{1}{2}}\beta$ of 127.7h. Radioactivity accumulated rapidly in tumours

**Figure 1** Toxicity of $^{131}$I-labelled MAb E48 in nude mice without xenografts monitored as the bodyweight relative to day 0, for diluent (○), 220μCi (●), 420μCi (▲), 670μCi (□) and 840μCi (■). Values are the mean of four mice per dose, standard deviations were less than 3%.

**Figure 2** Biodistribution of 800μCi $^{131}$I-labelled MAb E48 in nude mice bearing HNX-HN xenografts. Mice were bled, killed and dissected 2, 5 and 8h and 1, 3, 7, 10, 14, 21, 28 and 35 days after injection and the percentage injected dose per gram ($\%IDg^{-1}$) was calculated and plotted versus time. Tumour (●), blood (■) and lung (▲) are shown.

and reached a maximum of 19.4 ± 2.9% $IDg^{-1}$ after 3 days. Activity is retained in the tumour up to 8.1 ± 3.5% $IDg^{-1}$ at day 28. No specific accumulation is observed in any other tissue.

*Dosimetry calculations*

The absorbed cumulative radiation dose for tumour and various organs is shown in Figure 3. Based on the area under the curve of the biodistribution data of 800μCi $^{131}$I-labelled MAb E48 IgG the absorbed radiation dose to tumours in the group receiving 800μCi was 12,170cGy, whereas blood received only 2,984cGy. Other tissues received the following dose: lung; 662cGy; kidney; 607cGy; spleen: 581cGy; bladder: 571cGy; heart: 543cGy; colon: 424cGy; ileum: 405cGy; sternum: 405cGy; liver: 403cGy; muscle: 276cGy; stomach: 251cGy.

*Evaluation of therapeutic efficacy*

Tumour growth expressed as the tumour volume at each timepoint relative to the tumour volume at day 0 for control and treatment groups is shown in Figure 4. Tumours in the groups receiving unlabelled MAb E48 IgG (Figure 4a), 800μCi $^{131}$I-labelled control MAb IgG (Figure 4b) and diluent (Figure 4c) all showed exponential growth. Median tumour volume doubling times in the group receiving diluent or unlabelled MAb E48 IgG was 5.5 days. Median tumour volume doubling time in the group receiving 800μCi $^{131}$I-labelled control MAb showed a minimal, statistically insignificant increase. All tumours in the group receiving 400μCi $^{131}$I-labelled MAb E48 IgG (Figure 4d) showed delay of growth with a median tumour volume doubling time of 22.6 days, while two out of nine tumours showed regression. All tumours in the group receiving 800μCi $^{131}$I-labelled MAb E48 IgG (Figure 4e) showed regression, with a median tumour volume doubling time of 43 days. Moreover, in this group, two out of seven tumours showed complete remission without regrowth during follow-up (>3 months). After sacrificing these animals, no evidence of tumour could be detected at the site of implantation. The tumour growth delay factor calculated for the 400 and 800μCi groups was 3.1 and 6.8, respectively. Weight loss in experimental groups did not exceed 15% at any timepoint. When compared with the tumour growth delay factor of chemotherapeutic agents like adriamycin (3.0),5-fluorouracil(1.2), cisplatin (1.1), bleomycin (0.7), methotrexate (0) and 2',2'-difluorodeoxycytidine (1.6), established in the same HNX-HN xenograft, RIT shows a very high therapeutic efficacy (Figure 5). No cures were observed with chemotherapeutic agents. Pro-

**Figure 3** Total accumulated radiation dose in $10^3$cGy, calculated using the trapezoid integration method for the area under the curve. Tu, tumour; Blo, blood; Bla, bladder; Lu, lung; Ki, kidney; Sp, spleen; He, heart; Li, liver; Co, colon; Sto, stomach; Il, ileum; Ste, sternum; Mu, muscle.

**Figure 5** Antitumour effect of RIT in comparison with chemotherapy in HNX-HN xenografts. Antitumour effect was expressed as the tumour growth delay factor (see: Material and methods). DOX, doxorubicin; dFdC,2',2'-difluorodeoxcytidine; 5-FU, 5-fluorouracil; CP, cisplatin; BLEO, bleomycin; MTX, methotrexate (* = 0).

**Figure 4** Effects of unlabelled MAb E48, $n = 4$, $t = 5$ **a**, $^{131}$I-labelled control MAb, $n = 6$, /$t = 9$ **b**, diluent, $n = 3$, $t = 5$ **c**, 400µCi $^{131}$I-labelled MAb E48, $n = 6$, $t = 9$ **d**, and 800µCi $^{131}$I-labelled MAb E48, $n = 5$, $t = 7$ **e** on the growth of HNX-HN xenografts, expressed as the tumour volume during therapy relative to the tumour volume at the start of the therapy. Mice were sacrificed when tumours exceeded 1000mm$^3$, $n$ = number of animals, $t$ = number of tumours. * = complete remission without regrowth during follow up (>3 months).

longed survival, as determined by the Mann–Whitney U-test, was significant for both RIT groups as compared to control groups ($P$<0.01).

### Discussion

Therapeutic efficacy of radiolabelled MAbs in the nude mouse model has been described for several tumour types. Although clinical radioimmunoscintigraphy studies for the detection of HNSCC have been reported with $^{111}$In-labelled anti-epidermal growth factor receptor (Soo *et al.*, 1987) and with $^{111}$In-labelled anti-carinoembryonic antigen (Kairemo & Hopsu, 1990; Kairemo & Hopsu, 1990), no reports are available on therapy experiments of HNSCC xenografts with radiolabelled MAbs. Here we present the first data on RIT of HNSCC. As a first approach to assess the potential of radiolabelled MAb E48 in eradicating HNSCC xenografts, therapy experiments, consisting of single bolus injection of two different doses, were designed in a straight forward manner. Dosimetry calculations were based on the biodistribution of a therapeutic dose, since continued tumour growth in biodistribution experiments with tracer dose may well result in underestimation of the radiation dose up to 35–52% (Lee *et al.*, 1990; Badger *et al.*, 1986). In our studies, no differences in biodistribution between tracer and therapeutic doses were observed. In tracer dose studies however, no data were available during the first 12h and after day 7, whereas in this study data were obtained from 2h p.i. up to 35 days, allowing more accurate dosimetry calculations. A remarkable good retention of MAb E48 was observed with 8.1 ± 3.5%IDg$^{-1}$ at day 28 after injection.

Although numerous reports with $^{131}$I-labelled MAb IgG or F(ab')$_2$ have been described with anti-tumour effects, only few studies achieve complete remissions after single bolus injections. Wessels *et al.* reported complete remissions of renal cell carcinoma xenografts after single bolus injection of 600µCi $^{131}$I-labelled MAb IgG (Wessels *et al.*, 1989), whereas Sharkey *et al.* observed no regrowth of colon carcinoma xenografts after a single injection of 1mCi MAb IgG (Sharkey *et al.*, 1987). Lee *et al.* obtained apparent cures of mice with intracranial glioma xenografts after a single injection of 1.25mCi MAb IgG (Lee *et al.*, 1988). Complete ablation of highly radiation sensitive neuroblastoma xenografts was achieved with a single injection of 1mCi IgG by the group of Cheung *et al.* (Cheung *et al.*, 1986). Buchegger *et al.* completely eradicated xenografts of colon carcinomas with single injections of 2,200–2,800µCi, but instead of IgG, pooled F(ab')$_2$ fragments of three different anti-CEA MAbs were used (Buchegger *et al.*, 1989). Other successful studies applied fractionated protocols (Smith *et al.*, 1991; Senekowitsch *et al.*, 1989; Schlom *et al.*, 1990; Buchegger *et*

al., 1989). In our study, single injections of 400 or 800$\mu$Ci $^{131}$I-labelled E48 MAb IgG showed pronounced anti-tumour effects, resulting in complete remissions of two out of seven tumours in the group receiving 800$\mu$Ci $^{131}$I-labelled MAb E48 IgG. No remnant tumour could be detected when mice were sacrificed after 3 months follow-up. These cures might very well be due to the intrinsic sensitivity of head and neck tumours for radiation (Wessels et al., 1989a). In addition, the accumulated dose, 12,170cGy, in tumour tissue as a result of a single bolus injection 800$\mu$Ci was very high, reflecting the excellent targetting and retention characteristics of MAb E48 in this experimental model.

Therapeutic efficacy of RIT has been found to be inversely correlated with tumour size (Scholm et al., 1991; Lee et al., 1988; Sharkey et al., 1987). Accordingly, RIT has the potential to be the most useful in adjuvant therapy when minimal disease is present (Sharkey et al., 1987; Langmuir & Sutherland, 1988). In the case of head and neck cancer this would apply to patients with stage III and IV disease. In these patients local recurrences occur in 50–60%, while 15–25% develop distant metastases after surgery and/or radiotherapy (Choksi et al., 1988). Unfortunately, no relevant metastatic model for HNSCC is available. In our study, the correlation between tumour size and therapeutic effect could not be determined due to the selected size range.

In several studies, an increase in therapeutic efficacy combined with a decrease in toxicity has been observed when total dose was given in multiple fractions (Buchegger et al., 1990; Buchegger et al., 1989; Colapinto et al., 1990; Smith et al., 1991; Schlom et al., 1990). Therefore, the efficacy of RIT with MAb E48 with respect to growing, established HNX-HN xenografts will be further investigated comparing single injection regimen to multiple injection regimens. Furthermore, since $^{131}$I is not the isotope of choice in clinical applications because of the low percentage therapeutic $\beta$-emission (32%) and the high percentage damaging $\gamma$-radiation (66%), and because of the rapid dehalogenation of $^{131}$I-labelled conjugates, we have developed a MAb E48 radioimmunoconjugate labelled with $^{186}$Re, an isotope with a high percentage $\beta$-emission (90%) and low percentage $\gamma$-emission (8%). MAbs labelled with this isotope have already been described in tumour localisation and tumour therapy studies (Beaumier et al., 1991; Goldrosen et al., 1991). MAb E48 labelled with this isotope will be tested in the HNX-HN xenograft model.

Thusfar, clinical results with chemotherapy have been disappointing with respect to the effect on 5-year survival of patients, despite the number of trials over the past 10 years (Choksi et al., 1988; Snow, 1991). In our HNX-HN xenograft model, a number of conventional drugs, known to produce remissions in patients with head and neck cancer, and one experimental chemotherapeutic agent have been evaluated (Braakhuis et al., 1991) unpublished data). In the dose schedules described, none of the chemotherapeutic agents caused tumour growth delay factors higher than those obtained with either 800$\mu$Ci or 400$\mu$Ci $^{131}$I-labelled MAb E48 IgG. Furthermore, no cures were observed with these chemotherapeutic agents.

One of the limitations of the nude mouse xenograft model for RIT studies with radiolabelled MAbs is the absence of antigen expression in normal tissues. The presence of the MAb E48 defined antigen in normal tissues in the clinical situation will obviously influence the pharmacokinetics and biodistribution of radiolabelled MAb E48. In clinical radioimmunoscintigraphy studies using $^{99m}$Tc-labelled MAb E48 (Fab')$_2$ fragment we observed uptake of radioactivity in normal oral mucosa and adrenal glands (van Dongen et al., 1992). Uptake in these tissues seems to be diminished when using whole IgG. Most clinical trials with radiolabelled MAb for diagnosis or therapy of solid neoplasms have reported MAb uptake in large tumours in the range of 0.001–0.01%IDg$^{-1}$ (Goldenberg, 1991; Epenetos & Kosmas, 1989). Preliminary data on the localisation of $^{99m}$Tc-labelled MAb E48 IgG indicate accumulation of the conjugate in tumours of 0.5–4.0cm diameter up to a mean %IDg$^{-1}$ of 0.03 at 44h (range: 0.0143–0.0823, number of patients = 7). This looks very promising indeed, when taking into account the higher accumulation of MAbs in small tumour loads. Chatal et al. reported on the biodistribution of $^{111}$In-labelled MAb OC125 intraperitoneally injected into patients with ovarian carcinoma, demonstrating low accummulation in large tumours (0.0014–0.0032 %IDg$^{-1}$) but significantly higher accumulation in small tumour nodules ($0.13 \pm 0.08$ %IDg$^{-1}$) and malignant cell clusters (median 0.33 with a maximum of 4.16 %IDg$^{-1}$) (Chatal et al., 1989). Assuming that this size correlation also applies for head and neck tumours and assuming that patients will tolerate a dose of 100mCi of $^{131}$I-labelled MAB E48 (or an equivalent dose of $^{186}$Re-labelled MAb E48) (Rosen et al., 1987; Ward et al., 1988), achieving radiation doses in tumour tissue enabling elimination of minimal disease lies within reach.

Our data, showing the capacity of a single bolus injection $^{131}$I-labelled MAb E48 to eradicate HNSCC xenografts in nude mice, present the first successful RIT results for head and neck squamous cell carcinoma. Together with data from an ongoing phase I clinical trial in our hospital, showing the capacity of $^{99m}$Tc-labelled MAb E48 (F(ab')$_2$ fragment and IgG in detecting metastatic and recurrent disease, this indicates the potential of radiolabelled MAb E48 for radioimmunotherapy of patients with head and neck cancer.

This work was supported in part by Centocor Europe Inc., Leiden, The Netherlands.

### References

BADGER, C.C., KROHN, K.A., SHULMAN, H., FLOURNOY, N. & BERNSTEIN, I.D. (1986). Experimental radioimmunotherapy of murine lymphoma with $^{131}$I-labeled anti-T cell antibodies. *Cancer Res.*, **46**, 6223–6228.

BEAUMIER, P.L., VENKATESAN, P., VANDERHEYDEN, J.-L., BURGUA, W.D., KUNZ, L.L., FRITZBERG, A.R., ABRAMS, P.G. & MORGAN, A.C. Jr (1991). $^{186}$Re Radioimmunotherapy of small cell lung carcinoma xenografts in nude mice. *Cancer Res.*, **51**, 676–681.

BLUMENTHAL, R.D., KASHI, R., STEPHENS, R., SHARKEY, R.M. & GOLDENBERG, D.M. (1991). Improved radioimmunotherapy of colorectal cancer xenografts using antibody mixtures against carcinoembryonic antigen and colon-specific antigen-p. *Cancer Immunol. Immunother.*, **32**, 303–310.

BRAAKHUIS, B.J.M., VAN DONGEN, G.A.M.S., BAGNAY, M., VAN WALSUM, M. & SNOW, G.B. (1989). Preclinical chemotherapy on human head and neck cancer xenografts grown in athymic nude mice. *Head & Neck*, **11**, 511–515.

BRAAKHUIS, B.J.M., VAN DONGEN, G.A.M.S., VERMORKEN, J.B. & SNOW, G.B. (1991). Preclinical *in vivo* activity of 2'2'-difluorodeoxycytidine (Gemcitabine) against human head and neck cancer. *Cancer Res.*, **51**, 211–214.

BUCHEGGER, F., PELEGRIN, A., DELALOYE, B., BISCHOF-DELALOYE, A. & MACH, J.-P. (1990). Iodine-131-labeled MAb F(ab')2 fragments are more efficient and less toxic than intact anti-CEA antibodies in radioimmunotherapy of large human colon carcinoma grafted in nude mice. *J. Nucl. Med.*, **31**, 1035–1044.

BUCHEGGER, F., PFISTER, C., FOURNIER, K., PREVEL, F., SCHREYER, M., CARREL, S. & MACH, J.-P. (1989). Ablation of human colon carcinoma in nude mice by $^{131}$I-labeled monoclonal anti-carcinoembryonic antigen antibody F(ab')$_2$ fragments. *J. Clin. Invest.*, **83**, 1449–1456.

CHATAL, J.-F., SACCAVINI, J.-C., THEDREZ, P., CURTET, C., KREMER, M., GUERREAU, D., NOLIBE, D., FUMOLEAU, P. & GUILLARD, Y. (1989). Biodistribution of Indium-111-labeled OC125 monoclonal antibody intraperitoneally injected into patients operated on for ovarian carcinomas. *Cancer Res.*, **49**, 3087–3094.

CHEUNG, N.-K., LANDMEIER, B., NEELY, J., NELSON, A.D., ABRAMOWSKY, C., ELLERY, S., ADAMS, R.B. & MIRALDI, F. (1986). Complete tumor ablation with iodine-131-labeled disialoganglioside GD2-specific moncolonal antibody against human neuroblastoma xenografted in nude mice. *J. Natl Cancer Inst.*, **77**, 739–745.

CHIOU, R.K., VESSELLA, R.L., LIMAS, C., SHAFER, R.B., ELSON, M.K., ARFMAN, E.W. & LANGE, P.H. (1988). Monoclonal antibody-targeted radiotherapy of renal cell carcinoma using the nude mouse model. *Cancer*, **61**, 1766–1775.

CHOKSI, A.J., DIMERY, I.W. & HONG, W.K. (1988). Adjuvant chemotherapy of head and neck cancer: the past, the present and the future. *Seminars in Oncol.*, **15**, (suppl.), 45–49.

COGNETTI, E.V., PINNARO, P., CARLINI, P., RUGGERI, E.M., IMPIOBATO, F.A., ROSARIO DEL VECCHIO, M., GIANARELLI, D. & PERRINO, A. (1988). Neoadjuvant chemotherapy in previously untreated patients with advanced head and neck squamous cell cancer. *Cancer*, **62**, 251–261.

COLAPINTO, E.V., ZALUTSKY, M.R., ARCHER, G.E., NOSKA, M.A., FRIEDMAN, H.S., CARREL, S. & BIGNER, D.D. (1990). Radioimmunotherapy of intracerebral human glioma xenografts with 131I-labeled F(ab')2 fragments of monoclonal antibody Mel-14. *Cancer Res.*, **50**, 1822–1827.

DILMAN, L.T. (1969). Radionuclide decay schemes and nuclear parameters for use in radiation-dose estimation. In *The Society of Nuclear Medicine (MIRD Pamphlet No.4)*, New York, NY.

EPENETOS, A.A. & KOSMAS, C. (1989). Monoclonal antibodies for imaging and therapy. *Br. J. Cancer*, **59**, 152–155.

ESTEBAN, J.M., HYAMS, D.M., BEATTY, B.G., MERCHANT, B. & BEATTY, J.D. (1990). Radioimmunotherapy of human colon carcinomatosis xenograft with 90Y-ZCE025 monoclonal antibody: toxicity and tumor phenotype studies. *Cancer Res.*, **50**, 989s–992s.

GERRETSEN, M., QUAK, J.J., SUH, J.S., VAN WALSUM, M., MEIJER, C.J.L.M., SNOW, G.B. & VAN DONGEN, G.A.M.S. (1991). Superior localisation and imaging of radiolabelled monoclonal antibody E48 F(ab')2 fragment in xenografts of human squamous cell carcinoma of the head and neck and of the vulva as compared to monoclonal antibody E48 IgG. *Br. J. Cancer*, **63**, 37–44.

GOLDENBERG, D.M. (1991). Challenges to the therapy of cancer with monoclonal antibodies. *J. Natl Cancer Inst.*, **83**, 78–79.

GOLDROSEN, M.H., BIDDLE, W.C., PANCOOK, J., BAKSHI, S. Jr, VANDERHEYDEN, J.-L., FRITZBERG, A.R., MORGAN, A.C. & FOON, K.A. (1991). Biodistribution, pharmacokinetics, and imaging studies with $^{186}$Re-labeled NR-LU-10 whole antibody in LS174T colonic tumor-bearing mice. *Cancer Res.*, **50**, 7973–7978.

KAIREMO, K.J.A. & HOPSU, E.V.M. (1990*a*). Imaging of pharyngeal and laryngeal carcinomas with Indium-111-labelled monclonal anti-CEA antibodies. *Laryngoscope*, **100**, 1077–1082.

KAIREMO, K.J.A. & HOPSU, E.V.M. (1990*b*). Imaging of tumours in the parotid region with Indium-111-labelled monoclonal antibody reacting with carcinoembryonic antigen. *Acta Oncologica*, **29**, 539–543.

LANGMUIR, V.K. & SUTHERLAND, R.M. (1988). Dosimetry models for radioimmunotherapy. *Med. Phys.*, **15**, 867–873.

LEE, Y.-C.C., WASHBURN, L.C., SUN, T.T., BYRD, B.L., CROOK, J.E., HOLLOWAY, E.C. & STEPLEWSKI, Z. (1990). Radioimmunotherapy of human colorectal carcinoma xenografts using 90Y-labeled monoclonal antibody CO17-1A prepared by two bifunctional chelate techniques. *Cancer Res.*, **50**, 4546–4551.

LEE, Y., BULLARD, D.E., HUMPHREY, P.A., COLAPINTO, E.V., FRIEDMEN, H.S., ZALUTSKY, M.R., COLEM, R.E. & BIGNER, D.D. (1988*a*). Treatment of intracranial human glioma xenografts with $^{131}$I-labeled anti-tenascin monoclonal antibody 81C6. *Cancer Res.*, **48**, 2904–2910.

LEE, Y.-S., BULLARD, D.E., ZALUTSKY, M.R., COLEMAN, R.E., WIKSTRAND, C.J., FRIEDMAN, H.S., COLAPINTO, E.V. & BIGNER, D.D. (1988*b*). Therapeutic efficacy of antiglioma mesenchymal extracellular matrix $^{131}$I-labeled murine monoclonal antibody in a human glioma xenograft model. *Cancer Res.*, **48**, 559–566.

MULSHINE, J.L., CARRASQUILLO, J.A., WEINSTEIN, J.N., KEENAN, A.M., REYNOLDS, J.C., HERDT, J., BUNN, P.A., SAUSVILLE, E., EDDY, J., COTELINGAM, J.D., PERENTESIS, P., PINSKY, C. & LARSON, S.M. (1991). Direct intralymphatic injection of radiolabeled $^{111}$I-T101 in patients with cutaneous T cell lymphoma. *Cancer Res.*, **51**, 688–695.

QUAK, J.J., BALM, A.J.M., BRAKKEE, J.G.P., SCHEPER, R.J., HAISMA, H.J., BRAAKHUIS, B.J.M., MEIJER, C.J.L.M. & SNOW, G.B. (1989). Localization and imaging of radiolabelled monoclonal antibody against squamous cell carcinoma of the head and neck in tumor-bearing nude mice. *Int. J. Cancer*, **44**, 534–538.

QUAK, J.J., BALM, A.J.M., VAN DONGEN, G.A.M.S., BRAKKEE, J.G.P., SCHEPER, R.J., SNOW, G.B. & MEIJER, C.J.L.M. (1990*a*). A 22-kd surface antigen detected by monoclonal antibody E48 is exclusively expressed in stratified squamous and transitional epithelia. *Am. J. Pathol.*, **136**, 191–197.

QUAK, J.J., SCHRIJVERS, A.H.G.J., BRAKKEE, J.G.P., DAVIS, H.D., SCHEPER, R.J., BALM, A.J.M., MEIJER, C.J.L.M., SNOW, G.B. & VAN DONGEN, G.A.M.S. (1992). Expression and characterization of two differentiation antigens in stratified epithelia and carcinomas. *Int. J. Cancer*, **50**, 507–513.

QUAK, J.J., VAN DONGEN, G.A.M.S., GERRETSEN, M., HAYASHIDA, D., BALM, A.J.M., BRAKKEE, J.G.P., SNOW, G.B. & MEIJER, C.J.L.M. (1990*b*). Production of a monoclonal antibody (K 931) to a squamous cell carcinoma associated antigen identified as the 17-1A antigen. *Hybridoma*, **9**, 377–387.

ROSEN, S.T., ZIMMER, A.M., GOLDMAN-LEIKIN, R., GORDON, L.I., KAZIKIEWICZ, J.M., KAPLAN, E.H., VARIAKOJIS, D., MARDER, R.J., DYKEWICZ, M.S., PIERGIES, A., SILVERSTEIN, E.A., ROENIGK, H.H. & SPIES, S.M. (1987). Radioimmunodetection and radioimmunotherapy of cutaneous T cell lymphomas using an $^{131}$I-labeled monoclonal antibody: an Illinois Cancer Council Study. *J. Clin. Oncol.*, **5**, 562–573.

SCHLOM, J., MOLINOLO, A., SIMPSON, J.F., SILER, K., ROSELLI, M., HINKLE, G., HOUCHENS, D.P. & COLCHER, D. (1990). Advantage of dose fractionation in monoclonal antibody-targeted radioimmunotherapy. *J. Natl Cancer Inst.*, **82**, 763–771.

SCHLOM, J., SILER, K., MILENIC, D.E., EGGENSPERGER, D., COLCHER, D., MILLER, L.S., HOUCHENS, D., CHENG, R., KAPLAN, D. & GOECKELER, W. (1991). Monoclonal antibody-based therapy of a human tumor xenograft with a $^{177}$Lutetium-labeled immunoconjugate. *Cancer Res.*, **51**, 2889–2896.

SCHRIJVERS, A.H.G.J., GERRETSEN, M., FRITZ, J.M., VAN WALSUM, M., QUAK, J.J., SNOW, G.B. & VAN DONGEN, G.A.M.S. (1991). Evidence for a role of the monoclonal antibody E48 defined antigen in cell-cell adhesion in squamous epithelia and head and neck squamous cell carcinoma. *Exp. Cell. Res.*, **196**, 264–269.

SENEKOWITSCH, R., REIDEL, G., MOLLENSTADT, S., KRIEGEL, H. & PABST, H.-W. (1989). Curative radioimmunotherapy of human mammary carcinoma xenografts with iodine-131-labeled monoclonal antibodies. *J. Nucl. Med.*, **30**, 531–537.

SHARKEY, R.M., PYKETT, M.J., SIEGEL, J.A., ALGER, E.A., PRIMUS, F.J. & GOLDENBERG, D.M. (1987). Radioimmunotherapy of the GW-39 human colonic tumor xenograft with $^{131}$I-labeled murine monoclonal antibody to carcinoembryonic antigen. *Cancer Res.*, **47**, 5672–5677.

SMITH, A., GROSCURTH, P., WAIBEL, R., WESTERA, G. & STAHEL, R.A. (1990). Imaging and therapy of small cell carcinoma xenografts using $^{131}$I-labeled monoclonal antibody SWA11. *Cancer Res.*, **50**, (suppl.), 980s–984s.

SMITH, A., WAIBEL, R. & STAHEL, R.A. (1991). Selective immunotherapy of small cell cancer xenografts using $^{131}$I-labelled SWA11 antibody. *Br. J. Cancer*, **64**, 263–266.

SNOW, G.B. (1991). Head and neck: Editorial overview. *Current Opinion in Oncol.*, **3**, 497–499.

SOO, K.C., WARD, M., ROBERTS, K.R., KEELING, F., CARTER, R.L., MCREADY, V.R., OTT, R.J., POWELL, E., OZANNE, B., WESTWOOD, J.H. & GUSTERSON, B.A. (1987). Radioimmunoscintigraphy of squamous carcinomas of the head and neck. *Head & Neck Surgery*, **9**, 349–352.

STEWART, J.S.W., HIRD, V., SULLIVAN, M., SNOOK, D. & EPENETOS, A.A. (1989). Intraperitoneal radioimmunotherapy for ovarian cancer. *Br. J. Obstet. Gynaecol.*, **96**, 529–536.

van Dongen, G.A.M.S., Leverstein, H., Roos, J.C., Quak, J.J., van den Brekel, M.W.M., van Lingen, A., Martens, H.J.M., Castelijns, J.A., Visser, G.W.M., Meijer, C.J.L.M., Teule, J.J. & Snow, G.B. (1992). Radioimmunoscintigraphy of head and neck tumours using technetium-99m-labeled monoclonal antibody E48 $F(ab')_2$. *Cancer Res.*, **52**, 2569–2574.

Ward, B., Mather, S., Shepherd, J., Crowther, M., Hawkins, L., Britton, K. & Slevin, M.L. (1988). The treatment of intraperitoneal malignant disease with monoclonal antibody guided $^{131}I$ radiotherapy. *Br. J. Cancer*, **58**, 658–662.

Wessels, B.W., Harisiadis, L. & Carabell, S.C. (1989*a*). Dosimetry and radiobiological efficacy of clinical radioimmunotherapy. *J. Nucl. Med.*, **30**, 827.

Wessels, B.W., Vessella, R.L., Palme, D.F. II, Berkopec, J.M., Smith, G.K. & Bradley, E.W. (1989*b*). Radiobiological comparison of external beam irradiation and radioimmunotherapy in renal cell carcinoma xenografts. *Int. J. Radiation Oncol. Biol. Phys.*, **17**, 1257–1263.

Williams, J.A., Wessels, B.W., Edwards, J.A., Kopher, K.A., Wanek, P.M., Wharam, M.D., Order, S.E. & Klein, J.L. (1990). Targeting and therapy of human glioma xenografts *in vivo* utilizing radiolabeled antibodies. *Cancer Res.*, **50** (suppl.), 974s–979s.